# Postprandial Dysmetabolism and Its Medical Implications

**DOI:** 10.3390/life13122317

**Published:** 2023-12-10

**Authors:** Emanuel Sasso, Lara Baticic, Vlatka Sotosek

**Affiliations:** 1 Faculty of Medicine, University of Rijeka, Brace Branchetta 20, 51000 Rijeka, Croatia; 2 Department of Medical Chemistry, Biochemistry and Clinical Chemistry, Faculty of Medicine, University of Rijeka, Brace Branchetta 20, 51000 Rijeka, Croatia; 3 Department of Anesthesiology, Reanimatology, Emergency and Intensive Care Medicine, University of Rijeka, Braće Branchetta 20, 51000 Rijeka, Croatia; vlatkast@uniri.hr

**Keywords:** cancer, cardiovascular diseases, chronic low-grade inflammation, diabetes, obesity

## Abstract

An unbalanced diet increases the risk of developing a variety of chronic diseases and cancers, leading to higher morbidity and mortality rates worldwide. Low-grade systemic chronic inflammation mediated by the activation of the innate immune system is common to all these pathologies. Inflammation is a biological response of the body and a normal part of host defense to combat the effects of bacteria, viruses, toxins and macronutrients. However, when the innate immune system is constantly activated, it can promote the development of low-grade systemic chronic inflammation, which could play an important role in the development of chronic diseases and cancer. Since most chronic inflammatory diseases are associated with diet, a balanced healthy diet high in anti-inflammatory food components could prevent chronic diseases and cancer. The cells of the body’s immune system produce chemokines and cytokines which can have pro-inflammatory and tumor-promoting as well as anti-inflammatory and tumor-fighting functions. A challenge in the future will be to assess whether polymorphisms in immune-related genes may play a role in promoting pro-inflammatory activity. Thanks to this duality, future research on immune regulation could focus on how innate immune cells can be modified to convert a pro-inflammatory and tumor-friendly microenvironment into an anti-inflammatory and anti-tumor one. This review describes inflammatory responses mediated by the innate immune system in various diseases such as hyperglycemia and/or hyperlipemia, obesity, type II diabetes, cardiovascular disease and cancer.

## 1. Introduction

The postprandial state is known as the metabolic assessment period during and after a meal (6–12 h), which involves the digestion and absorption of nutrients, mainly fatty acids and carbohydrates from food. This state spans most of the day, more than 16 h, and is characterized by an increase in glycemia and lipidemia associated with systemic low-grade inflammation [1]. Inflammation is an essential component of innate (nonspecific) immunity and host defense, but a chronic systemic low-grade inflammatory state is also the basis of the metabolic syndrome.

The modern diet causes a state of chronic systemic low-grade inflammation due to its unbalanced composition, which includes an increased consumption of red meat, glucose and fatty acids and a low intake of fruits and vegetables. Pro-inflammatory factors in today’s diet include the consumption of saturated fatty acids and industrially produced fatty acids, a high ratio of omega-6 to omega-3 fatty acids, the low intake of omega-3 long-chain polyunsaturated fatty acids from fish, foods with a high glycemic load and the consumption of carbohydrates with a high glycemic index, and the low status given to vitamins and minerals [2]. Eating habits have changed drastically, such as the consumption of high amounts of red meat from fast food restaurants and convenience foods high in fat and glucose [3]. This type of diet is known to increase the risk of diseases such as obesity, metabolic-dysfunction-associated steatotic liver disease (NAFLD), type 2 diabetes mellitus, cardiovascular disease, certain types of cancer (e.g., breast, colon and pancreatic cancer) and neurodegenerative disease (e.g., Alzheimer’s disease) [1,2,3] (Table 1). All of these pathologies have escalated in the 21st century, replacing infectious diseases as the leading cause of mortality, and today they are the leading public health problem worldwide, especially in developed countries [1,2,3,4,5].

**Figure 1:**
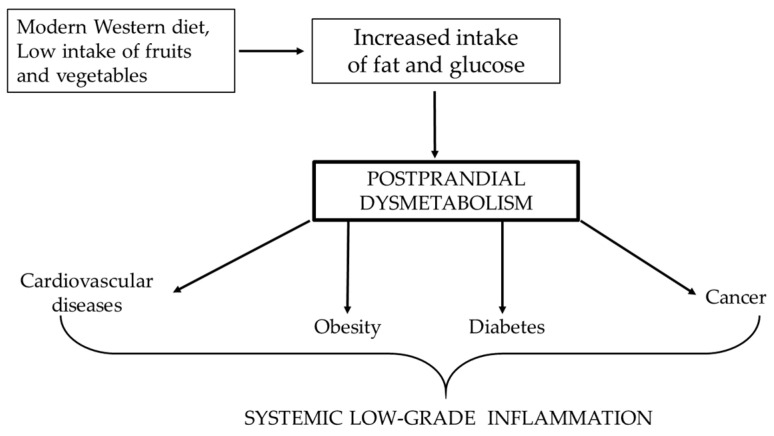
Correlation between Western diet and the increased risk of pathologies, of which the lowest common denominator is systemic low-grade inflammation.

The unifying element of all these diseases is chronic systemic low-grade inflammation (Figure 1), caused by high glucose levels and free fatty acids, which trigger the production of free radicals and induce the release of proinflammatory cytokines such as interleukin-6 (IL-6) and tumour necrosis factor α (TNFα) [2,3,4,5,6]. Therefore, the aim of this review was to comprehensively analyze and summarize the correlation between aspects of the postprandial state, chronic systemic low-grade inflammation and the leading pathologies of the modern era.

**Figure 2:**
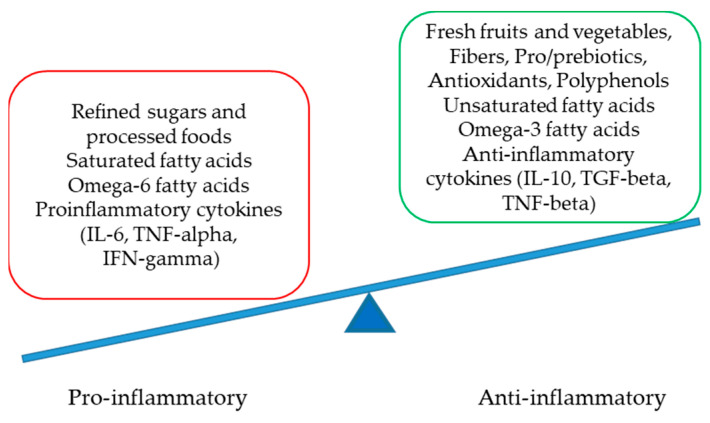
The delicate equilibrium between pro- and anti-inflammatory statuses (IL-interleukin, TNF-tumor necrosis factor, IFN-interferon and TGF-transforming growth factor).

Numerous factors are involved in the delicate but complex equilibrium between pro- and anti-inflammatory statuses at the molecular level. Crucial players are presented in Figure 2.

## 2. Hyperglycemia, Hyperlipidemia and the Inflammatory Response

**Figure 3:**
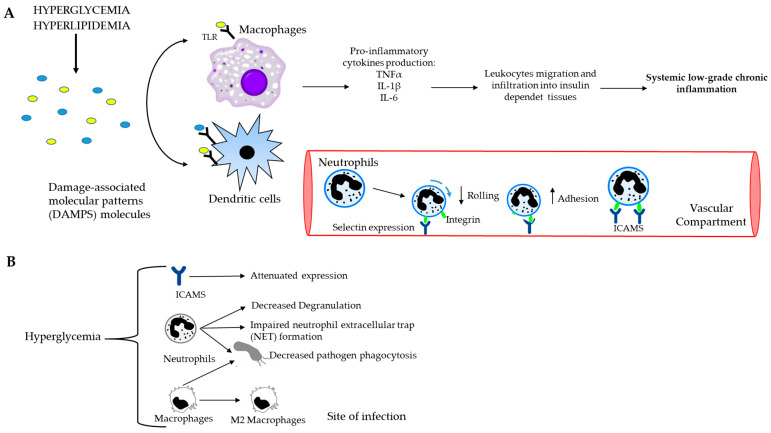
Causal connection between the immune system and postprandial dysmetabolism. (**A**) Systemic low-grade chronic inflammation starts with macrophages and dendritic cells, which recognize and bind DAMP molecules through toll-like receptors (TLRs). Consequently, the production of pro-inflammatory cytokines and the recruitment of immune cells to the site of the damage occur. The further spreading of the leukocytes towards insulin-dependent tissues leads to chronic inflammation. (**B**) Schematic representation of hyperglycemia and innate immune cells. High blood glucose level decreases rolling ability and increases the adhesion capacity of neutrophils to endothelium, impairs NET formation and decreases phagocytosis of both neutrophils and macrophages.

Inflammation can be defined as a biochemical response to cell injury and/or stress, which manifests in increased blood flow, capillary dilation, leukocyte infiltration and the attraction of monocytes, which are directed to the site of the injured tissue by chemotactic factors and differentiate into macrophages. The latter are the main source of growth factors and cytokines that influence endothelial, epithelial and mesenchymal cells in the microenvironment to eliminate toxic substances and promote the repair of damaged tissue [7]. Injury-induced tissue damage produces molecules called damage-associated molecular patterns (DAMPs) that are recognized by sentinel cells, such as macrophages and dendritic cells, via toll-like receptors on their membranes (Figure 3A). This injury-induced tissue damage triggers a complex sequence of events leading to an inflammatory response determined by a local increase in blood flow and capillary permeability with the activation of the endothelial inducible form of nitric oxide synthase, the efflux of plasma proteins and the sequestration of immune cells, leading to lysis and the restoration of physiologic conditions. Indeed, hyperglycemia leads to a rapid and strong increase in adhesion molecules on monocytes and neutrophils, causing the integrin-receptor-mediated adhesion of these cells to the endothelium. In addition, the exposure of endothelial cells to high glucose concentrations leads to an increased expression of adhesion molecules, such as endothelial leukocyte adhesion molecule-1, vascular cell adhesion molecule-1 and intercellular adhesion molecule-1 [8].

Complement system activation, which is related to blood glucose levels, plays a crucial role in the early inflammatory process of the innate immune defense. The complement system is activated during and after a meal, with chylomicrons in particular being responsible for the activation of complement factor 3 (C3). When fatty acids are consumed and digested, they are incorporated into chylomicrons in the intestine, which then release lipids into muscle and adipose tissue. More specifically, adipose tissue is the site where C3 is activated by chylomicrons, and this increase in C3 levels is associated with postprandial lipemia in humans. However, in the fasting state, increased C3 levels are associated with insulin resistance, obesity and coronary heart disease. In summary, C3 is important for the excretion of fats but can lead to low-grade inflammation when overeating [1]. Furthermore, complement components and certain cytokines are responsible for the promotion of the procoagulant state under hyperglycemic conditions, especially interleukin 6 (IL-6), activating immune and endothelial cells. In fact, it has been reported that pro-inflammatory cytokines (e.g., TNFα, interleukin 1β (IL-1β), interleukin 18 (IL-18) and IL-6) are increased in acute hyperglycemia, whereas in the opposite condition, when insulin is working, these cytokines are significantly decreased. Distinctly, TNFα is able to induce insulin resistance by reducing the messenger RNA expression for glucose transporter 4 (GLUT4), a trans-membrane channel for glucose uptake into peripheral tissues, and to mobilize lipids from adipocytes, decreasing lipoprotein lipase activity. Acute hyperglycemia is a condition in which pro-inflammatory cytokines dominate the innate immune response during the early phase [8].

One of the cellular components of the innate immune response are neutrophils, which are recruited to the site of injury to eliminate pathogens. Under conditions of high glucose, physiologic homeostasis is altered in several ways: neutrophils migrate, as they have an increased adherence capacity to the endothelium; phagocytosis occurs and the bactericidal activity of neutrophils changes, as the high glucose level inhibits the formation of neutrophil extracellular traps (these are smooth filaments of chromatin and various cytoplasmic proteins that, in combination with elastases and the enzyme myloperoxsidase, are involved in the clearance of pathogens, whose numbers are reduced); and Toll-like receptors are downregulated. These receptors are expressed by the membrane of neutrophils and have the task of increasing the production of cytokines and phagocytosis [8,9].

In addition, a diet high in fat and carbohydrates not only leads to inflammatory reactions but also to the formation of reactive oxygen species (ROS), which leads to an increase in oxidative stress [1]. Moreover, a high-fat diet leads to metabolic endotoxemia. This condition refers to a high concentration of lipopolysaccharide (LPS) in the blood, which is produced by the gut microbiota and contributes to the development of inflammatory biomarkers such as IL-6 and the soluble endotoxin receptor sCD14. Last but not least, the diversity of the gut microbiota also changes depending on one’s diet. Indeed, a high-fat diet in mice leads to a decrease in *Lactobacillus* spp., *Bacteroides*–*Prevotella* spp. and *Bifidobacterium* spp., an increase in intestinal permeability and an increase in circulating LPS and inflammatory markers [1,3,6].

## 3. Obesity and Inflammation

Obesity is known as an accumulation of adipose tissue in the body, mostly due to continuous overeating, as a result of consuming high-calorie foods. Hyperadiposity leads to chronic low-grade inflammation and an increased risk of developing insulin resistance, type 2 diabetes, fatty liver disease, atherosclerosis and cancer [10]. This type of inflammation in adipose tissue is a key feature of obesity; as a result of adipocyte hypertrophy, cytokines and chemokines are produced from the cells themselves and hypoxic areas are formed. The latter depend on the hypertrophy of the fat cells, which compress the blood vessels and thus reduce the oxygen supply to the vessels, leading to the necrosis of the fat cells. Therefore, the recruitment of macrophages and the expression of pro-inflammatory cytokines from adipocytes, immune cells and necrotic cells determine the inflammatory state. In addition, the necrotic debris resulting from adipocyte death is released into the extracellular space and exacerbates the inflammatory response [10,11]. To better understand this complex inflammatory process, it is crucial to identify two types of macrophages: macrophages of the M1 phenotype, which are involved in inflammation by producing pro-inflammatory cytokines, such as IL-1β, IL-6 and TNFα; and macrophages of the M2 phenotype, which are responsible for the production of anti-inflammatory cytokines, like interleukin 10 (IL-10) [11].

In those who have obesity, an increase in macrophages reminiscent of M1-polarized macrophages with pro-inflammatory activity that release cytokines such as TNFα was found. In addition, the ratio between M1 and M2 increases, and this is a marker of metabolic disease, which plays a role in the development of insulin resistance [12]. Macrophages are not the only players in this condition; lymphocytes are also involved in this process. Normally, different types of immune system cells that control and regulate the hormonal sensitivity of fat cells are located in the fatty acid stores, mainly represented by Th2 lymphocytes, which monitor the health status of the tissue and its metabolism. In addition, they release cytokines to regulate eosinophils and mast cells and to maintain macrophages in a polarized M2 state. These macrophages in turn produce molecules such as IL-10 to maintain the insulin sensitivity of fat cells [13].

**Figure 4:**
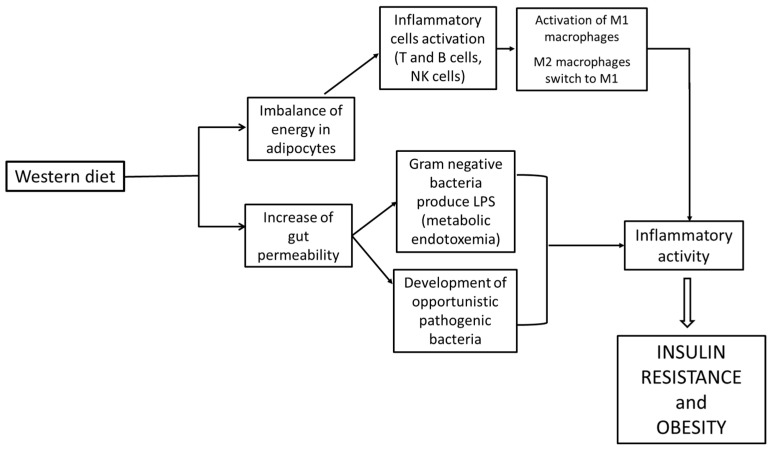
Correlation between Western diet, inflammation and obesity.

Th1 lymphocyte activation has been reported in obesity, as occurs in infection; lymphocytes’ T effector cells, plasma B cells and natural killer cells (NK cells) stimulate and activate M1 macrophages in adipose tissue (Figure 4) [12]. In the adipose tissue, macrophages are maintained in the M2 state by eosinophils and natural helper lymphocytes through the production of cytokines like interleukin IL-4 to prevent the development of obesity [14]. The suppression of CD4+ T regulatory cells (CD4+ Tregs) occurs in obesity as well [15].

Another critical factor that triggers inflammation is the imbalance of energy in adipocytes and the increase in gut permeability, as Gram-negative bacteria produce lipopolysaccharide (LPS), which leads to an inflammatory response by binding to TLR-4 receptors located on macrophages and adipocytes [16,17].

Inflammation in obese patients leads to insulin resistance due to the chronic inflammation triggered by obesity [10]. In this process, as previously shown, immune cells reach metabolic organs, such as the liver and adipose tissue, and release proinflammatory cytokines, such as TNFα and IL-6 [18]. TNFα activates the phosphorylation of tyrosine in the insulin resistance (IR) and insulin receptor substrate molecule (IRS) signaling pathways by TNFα-induced kinases, such as c-Jun N-terminal kinase or IkB kinase, which impede the progression of the insulin pathway [19,20]. IL-6 originates from the stromal vascular portion of visceral adipose tissue, which enters the liver via the portal vein and binds to the α-chain of the IL-6 receptor and the glycoprotein GP130, which are localized on the membrane of hepatocytes and immune cells. IL-6 can also come into contact with other cells that do not possess these receptors via trans-signaling. In both cases, the signaling pathway begins with Janus kinase (JAK2/STAT3), which activates the gene transcription of SOCS3 [18]. The ubiquitination and degradation of insulin receptor substrate 1 (IRS1) locks the insulin signal transduction at the IRS protein level [21].

Recent scientific studies have shown a close link between obesity and gut microbiota [22]. In obese individuals, there is an altered composition of the gut microbiota, which determines a reduction in the protective barriers formed by microorganisms such as *Lactobacillus* and *Bifidobacterium*, in association with the increase in opportunistic pathogenic bacteria, such as *Enterobacteriaceae*, *Streptococcus* and *Desulfovibrionaceae*. These data have been found in several studies in both mice and humans; there is a 50% increase in *Firmicutes* and a 50% decrease in *Bacteroidetes* in obese mice susceptible to high-fat diets or genetic obesity, and a high ratio of *Firmicutes* to *Bacteroidetes* has been found in obese humans [23,24]. De Filippo et al. described how diet can influence the composition of gut microbiota [25]. In their work, European children’s fecal microbiota, coming from a sample of young individuals living in the urban area of Florence (Italy), were compared to children’s fecal microbiota in Burkina Faso. This work underlines the clear difference in the ratio between *Firmicutes* and *Bacteroidetes phyla* and shows that in European children, this ratio is high, with more *Firmicutes* than *Bacteroidetes*, while children in Burkina Faso have a low ratio. Thus, this relation is considered a predisposition to obesity.

The difference between these two groups lies in their different dietary lifestyles; in fact, Europeans tend to consume a typical Western diet based on animal proteins, sugar, starch, fatty acids and a small amount of vegetable fiber, whereas the diets of the people from Burkina Faso comprise more vegetable fibers, light fatty acids and animal proteins. It should be emphasized that in high-fat diets, there is an increase in Gram-negative bacteria, in combination with increased levels of LPS and a reduction in the permeability of the intestinal epithelium, due to higher levels of interferon γ (IFN-γ) and IL-1β, which have a negative impact on gut homeostasis [26].

## 4. Type 2 Diabetes and Postprandial Dysmetabolism

### 4.1. General Considerations

*Diabetes mellitus* (DM) is a chronic metabolic disease characterized by hyperglycemia resulting from defects in insulin secretion, insulin action or both. Chronic hyperglycemia due to a lack of insulin production or insulin resistance is associated with long-term damage, dysfunction and the failure of different organs and is a severe risk factor for various pathologies, such as cardiovascular, neurological and kidney diseases, among others [27]. DM is classified into two types, type 1 diabetes (T1D) and type 2 diabetes (T2D), the latter of which will be examined in this review. T2D is marked by insulin resistance and the productivity dysfunction of insulin secreted from β cells of the pancreas. These conditions are linked to smoking, aging, obesity and an incorrect lifestyle. In fact, patients suffering from T2D have high glucose levels and lipids in their blood which are indicative of a postprandial dysmetabolism condition [28].

Under physiological conditions, the release of insulin produced by β cells of the pancreas follows the glycemic curve during a meal with the purpose of reducing excessive plasma glucose levels, and it is accompanied by an oxidative and inflammatory response [29]. The insulin production in T2D patients does not grow proportionally to the glycemic load but is delayed, leading to higher and longer postprandial glucose excursions. Thus, the liver and peripheral tissues are not able to stimulate the uptake of glucose [30]. This condition also affects T2D patients’ lipid metabolism, as they have high levels of very-low-density lipoproteins (VLDLs), chylomicrons (CMs) and chylomicron remnants (CMRs) and low levels of high-density lipoproteins (HDLs) [31]. The increase in VLDLs is associated with the production of low-density LDLs, a subset of LDLs with a high triglyceride content that leads to an atherogenic risk. These conditions, along with insulin resistance, impair the uptake of fatty acids into muscle and lead to an increase in postprandial fatty acids in patients with T2D [32,33]. In the following sections, the features of T2D, the factors involved in this pathology, inflammation and its interactions with other organs are analyzed.

### 4.2. Insulin Resistance and Inflammation

The release of postprandial insulin, which is produced and released by β cells of the pancreas, is also controlled by incretins such as glucagon-like peptide 1 (GLP1) and glucose-dependent insulinotropic polypeptide (GIP) [34]. Normally, GLP1 and GIP are produced by the gastrointestinal tract during a meal, and their function consists of reducing the postprandial lipid concentration. T2D causes a low level of postprandial GLP1 together with an impairment of other incretin effects, leading to a high glucose peak after a meal [34,35].

As written above, insulin resistance is one of the main steps that lead to the development of T2D and it is associated with obesity, a lack of physical activity and aging; in T2D patients, in fact, insulin is not sufficient to act against the high level of glucose consumed during a meal [27,36]. Key players that were found to be involved in insulin resistance are inflammatory cytokines like TNFα, IL-6, C-reactive protein and plasminogen activator inhibitor, all of which were found to be elevated in obese mice [37,38]. These molecules, together with ROS and free fatty acids, activate Ikβα kinase β (IKKβ) and c-Jun N-terminal kinase 1 (JNK-1) which phosphorylate the serine residues of IRS protein and inhibit insulin signaling in liver and adipose tissue. In addition, IKKβ phosphorylates Ikβα and promotes its ubiquitination and degradation. During this process, it stimulates nuclear factor (NFkβ) translocation into the nucleus to activate the transcription of inflammatory genes [39].

**Figure 5:**
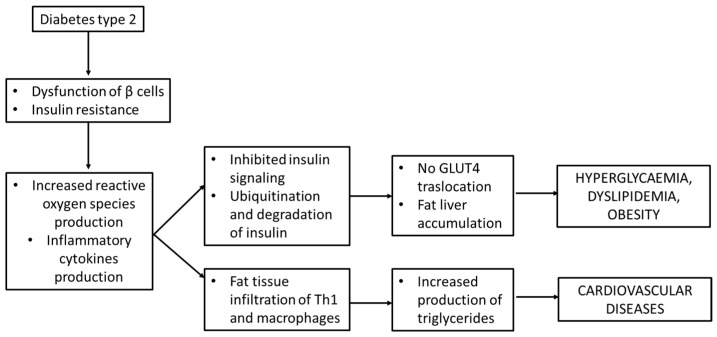
Insulin resistance and inflammation in *diabetes mellitus* type 2.

The inhibition of insulin signaling may also impair the Glut-4 translocation to cell membranes, thus leading to hyperglycemia, through Janus kinase/signal transducers and the activator of transcription (JAK/STAT) pathway. As described by Ye J. et al., the perpetrator of JAK/STAT activation is JNK. JAK phosphorylates the tyrosine of STAT and then induces the dimerization and translocation of STAT to the nucleus that phosphorylates the serine residues on IRS-1 [40]. All these processes lead to hyperglycemia but not the translocation of GLUT-4 to cell membranes [39] and are schematically presented in Figure 5.

### 4.3. Inflammation and Islets of Langerhans Failure

Pancreatic islets are composed of two compartments: the first one for the production of insulin and the second one made up of innate immune cells. Among the latter, macrophages do not follow the M2/M1 configuration like in adipose tissue, but they are found only in the M1 state, which occurs in healthy islets with a high expression of IL-1β, TNFα and the pro-inflammatory transcription factor interferon regulatory factor (IRF)-5 [41,42]. It seems that the function of macrophages is to monitor and enhance insulin secretion of β cells through endogenous ATP co-produced with insulin and the production of factors like retinoic acid [43,44].

The chronic state of obesity triggers negative conditions like hyperglycaemia and T2D, due to the insufficient production of insulin. This phenomenon is called “β cells failure” and consists of an inflammatory process, with the production of pro-inflammatory cytokines such as IL-1β, TNFα and chemokines ligand 2 (CCL-2). In addition, there is an increase in macrophages, which occurs in both humans affected with T2D and mice with diet-induced or genetically induced obesity [45].

Recent studies confirm that macrophages release IL-1β under the stimuli of endocannabinoids, amyloid polypeptide and free fatty acids. In particular, the latter initially stimulates β cells to produce IL-1β and pro-inflammatory cytokines. Simultaneously, this phenomenon brings about an augmentation of nitric oxide levels as a result of the reduction in mitochondrial ATP. This event leads to β cell dysfunction and to a decreased production of insulin [45,46].

Hyperglycemia, in combination with insulin deficiency, leads to an impairment of both innate and adaptive immune responses. First, the production of cytokines, such as IL-1β, interleukin 2 (IL-2) and IL-6, produced by peripheral blood mononuclear cells is reduced under high glucose conditions, causing a decreased action against pathogens [39]. Secondly, hyperglycemia is responsible for the impairment in the recruitment of leukocytes and CD8+ T cells in mice since the adhesion molecules seem to be less expressed. Third, the properties neutrophils, such as their opsonization, phagocytosis, NET traps and ROS production, are decreased. Fourth, macrophages have a reduced phagocytosis due to the impairment of complement receptors and Fcγ receptors on isolated monocytes, changing their phenotype in M2 in mice, which causes a weak immune response against pathogens [39].

## 5. Relation between Type 2 Diabetes and Obesity

Recent scientific and clinical studies show that obesity increases the risk of developing T2D, with an incidence seven times higher than in normal-weight individuals [47]. The common element connecting these two diseases is low-grade chronic inflammation, which determines the presence of inflammatory molecules, such as C-reactive protein, TNFα and IL-6 [48]. This phenomenon is referred to as meta-inflammation and develops through insulin resistance in the liver, skeletal muscle, adipose tissue and pancreas. In the liver, there is an accumulation of fats that precedes T2D and is related to obesity; namely, the reduction in hepatic insulin sensitivity leads to hyperglycemia. In addition, bad habits such as low physical activity and high-calorie diets lead to an increase in free fatty acids in the liver as well as in all the organs mentioned above, causing β-cell dysfunction [45,48].

The liver is the organ most affected by insulin resistance as it plays an important role in glucose homeostasis; this is this case in non-alcoholic fatty liver disease (NAFLD) in obese and T2D patients with hyperinsulinemia, hyperglycemia and dyslipidemia. This is the result of impaired lipid metabolism, in which lipid accumulation leads to mitochondrial dysfunction and subsequently triggers non-alcoholic fatty steatohepatitis (NASH) [49,50]. The NAFLD associated with insulin resistance consists of two main aspects: (1) the adipose tissue produces pro-inflammatory cytokines, such as TNFα, IL-1β and IL-6, that reduce the liver insulin sensitivity and consequently create an inflammatory phenomenon; and (2) the liver lipid accumulation causes mitochondrial dysfunction and the infiltration of immune system cells like recruited hepatic macrophages (RHMs), natural killer cells and T cells. In particular, RHMs are pro-inflammatory macrophages that produce chemokines for the recruitment of other monocytes into the liver [51,52]. These two aspects lay the foundations for NASH and later for hepatic cirrhosis and carcinoma. Moreover, the release of TNFα and IL-1β provoke M2 vs. M1 polarization, and this is the distinctive feature of metaflammation [51].

White adipose tissue and visceral adipose tissue play an important role in the inflammatory process in T2D patients as they produce proinflammatory cytokines, such as TNFα, IL-1 and IL-6; hormones such as leptin and adiponectin; and macrophage infiltration, which promotes local and chronic subclinical inflammation leading to T2D and cardiovascular disease [46]. Several studies show that in adipose tissue, there is also the infiltration of subpopulations of Th1 and Th2 lymphocytes. In T2D, there is a disturbance in the ratio between Th1/Th2 in favor of the first, which produces cytokines such as INFγ and TNFα and suppresses Th2 with its Tregs cytokines; this phenomenon is caused by insulin resistance and oxidative stress and leads to coronary artery disease [53]. Some research illustrates that the cell’s energetic compartment, constituted by the mitochondria, plays an important role in adipocyte homeostasis. In particular, the dysfunction of mitochondria contributes to insulin resistance and inflammation; in fact, it stimulates the augmentation of triglycerides and reduces the lipids’ oxidation [43]. In addition, in obese and T2D subjects, a reduction in adiponectin and simultaneously an increase in leptin have been found. In normal conditions, adiponectin is well known for its anti-inflammatory and anti-atherogenic features and it may also stimulate the release of insulin [43]. Thus, in both obese and T2D patients, low levels of adiponectin and high levels of leptin have been reported. The effects of leptin can be described as follows: (1) it reduces insulin secretion; (2) increases hepatic lipids; and (3) stimulates glucose uptake and oxidation in skeletal muscle. Moreover, leptin contributes to the pro-inflammatory effect on T cells and to the production of inflammatory cytokines like TNFα and IL-6 [54,55].

In chronic inflammation triggered by postprandial dysmetabolism, adaptive immunity, specifically T cells, plays a significant role in orchestrating and perpetuating the inflammatory response [1,39]. Postprandial dysmetabolism, characterized by fluctuations in glucose and lipid metabolism following meals, can lead to sustained low-grade inflammation. The involvement of adaptive immunity, particularly T cells, in this process involves several key mechanisms, like the activation and infiltration of T cells and the production of proinflammatory cytokines by activated T cells (IL-6, TNF- α and IFN-gamma) [8,26]. These cytokines contribute to sustaining the inflammatory environment, promoting insulin resistance and disrupting metabolic homeostasis. In addition, dysregulated lipid and glucose metabolism can generate neoantigens or modified self-antigens that are recognized by T cells, initiating adaptive immune responses that contribute to chronic inflammation associated with postprandial dysmetabolism [39,53]. The coordination between adaptive immunity (T cells) and innate immunity in postprandial dysmetabolism involves a complex interplay of immune cells and signaling molecules, such as macrophages and dendritic cells which secrete pro-inflammatory cytokines and chemokines that contribute to the recruitment and activation of T cells, amplifying the inflammatory response (Figure 5). As a result, the sustained inflammatory environment contributes to metabolic dysfunction, insulin resistance and tissue damage [1,8,39].

The skeletal muscle is also involved in the phenomenon of insulin resistance. The involvement of skeletal muscle in insulin resistance and reduced cellular responsiveness to insulin is significantly relevant to understanding the pathophysiology of metabolic disorders, particularly type 2 *diabetes mellitus* [28,29,30]. Insulin prompts glucose uptake into skeletal muscle cells by facilitating the translocation of glucose transporter proteins, predominantly GLUT4, to the cell membrane. In instances of insulin resistance, this process becomes compromised, deterring efficient glucose uptake by muscle cells. Moreover, insulin initiates intracellular signaling cascades upon binding to its receptor on muscle cells, triggering events that regulate glucose metabolism, which is altered in cases of insulin resistance. Elevated levels of intramuscular lipids, including fatty acids and triglycerides, are associated with insulin resistance as well [56]. The lack of mitochondrial function, the impairment of lipid storage in the muscle cells, the reduction in oxidative capacity and difficulties with the secretion of adiponectin represent major factors in obesity [56,57]. Excessive lipid deposition within skeletal muscle disrupts insulin signaling pathways, contributing to diminished insulin sensitivity. Dysfunction in skeletal muscle mitochondria, responsible for cellular energy production, is linked to insulin resistance as well. Impaired mitochondrial function destabilizes the cell’s capacity to efficiently metabolize glucose, contributing to metabolic disturbances. Persistent low-grade inflammation and oxidative stress in skeletal muscle detrimentally affect insulin signaling pathways, promoting insulin resistance, while inflammatory cytokines and reactive oxygen species (ROS) obstruct insulin action within muscle cells. Genetic predisposition and epigenetic modifications can impact skeletal muscle metabolism and insulin sensitivity as well, which can contribute to susceptibility to insulin resistance [52].

It has been demonstrated that in skeletal muscle, there are cytokines called myokines like irisin, myonectin and myostatin, but there have not been many scientific findings regarding the role of myokines in T2D and obesity. The best hypothesis is that these cytokines do not contribute directly to inflammation, but the infiltration of macrophages and T cells are the main actors that, together with high-calorie diets, lead to insulin resistance [57]. In particular, studies confirm that skeletal muscle also contains some macrophages in the intermyocellular/intermuscular adipose tissue between muscle fibers and in the periphery of muscles. These cells are pro-inflammatory and have the M1 phenotype, which in combination with lymphocyte Th1, CD4^+^ and CD8^+^ T cells, contribute to inflammation and insulin resistance [52].

Lifestyle changes emphasizing regular exercise, healthy dietary habits and weight control play fundamental roles in optimizing skeletal muscle function and fighting insulin resistance. Furthermore, targeted therapeutic approaches focusing on improving skeletal muscle metabolism may hold promise in addressing insulin resistance and related metabolic conditions.

## 6. Cardiovascular Diseases and Postprandial Dysmetabolism

Nowadays, the relationship between metabolic status and cardiovascular diseases is well known. Alterations in glucose and lipid homeostasis are the most important risk factors for cardiovascular disease, as postprandial hyperglycemia and hyperlipemia characterize postprandial dysmetabolism. This is the pathway that leads to atherosclerosis and cardiovascular damage through endothelial dysfunction [57].

The first cause of CVD is endothelial dysfunction, in which excess nutrients lead to electron chain overload in muscle and adipose tissue mitochondria, resulting in increased ROS production. High triglyceride levels are associated with a decrease in HDL and there is a resulting increase in VLDLs, which can enter the vascular wall, where the triglycerides are trapped by extracellular structures and cause lipid accumulation. This results in the formation of atherogenic plaques, which determine the development of atherosclerosis [29,58].

Inflammation is the second element involved in the process of atherosclerosis. Due to postprandial hyperglycemia and hyperlipidemia, there is an increase in circulating pro-inflammatory cytokines (IL-1β, TNFα and IL-6) in the blood, which originate from adipose tissue and connective tissue cells and promote chronic low-grade inflammation. In addition, other inflammatory components such as the NOD-like receptors of the inflammasome (NLRP3) contribute to the increasing inflammatory state [59,60].

Molecules such as cytokines (TNFα), cholesterol crystals and oxidized LDL are able to bind the pattern recognition receptors (PRR) on the cell membrane (macrophages and dendritic cells) and, by oligomerizing NLRP3 and activating the caspase-1 enzyme, they lead to the production of proinflammatory cytokines (IL-1β and IL-18) that stimulate inflammation, the expression of adhesion molecules of the endothelium and the increase in ROS production. In particular, the high concentrations of reactive oxygen species are not only responsible for the damage to the endothelial basement membrane as a result of the release of metalloproteases, but they also play an important role in the formation of the aforementioned oxidized LDL. The combination of both processes contributes to the development of plaque [61]. A third important element of vascular damage is hemostatic changes due to hyperglycemia and hyperlipidemia, which increase the risk of thrombotic events. Abnormal blood glucose levels increase platelet activity, and it appears that this event is caused by the upregulation of pro-aggregatory factors, such as P-selectin, thromboxane A2 and von Willebrand factors, which stimulate platelet aggregation and adhesion. Hyperglycemia is also responsible for the decrease in membrane fluidity due to the glycation of its membrane proteins, leading to the increased uptake of calcium, which in turn favors platelet activation and aggregation. In addition, hyperlipidemia leads to an increase in factor VIIc and factor VIIa and a decrease in plasminogen activator inhibitors, which leads to further platelet aggregation and activation [62,63].

## 7. Cancer, Inflammation, Nutrition and Gut Microbiota

Many studies point to an indirect link between diet and cancer; overeating and unbalanced diets lead to chronic inflammation mediated by innate immunity, which influences cancer development. Tissue damage and subsequent cell proliferation to repair tissues induced by chronic inflammation favor genomic instability and cancer development [1,6,64]. The chronic inflammatory microenvironment is mainly characterized by macrophages and leukocytes that produce high levels of reactive oxygen and nitrogen species to fight infections. The latter can also be generated by a locally increased metabolism. Reactive oxygen and nitrogen species produce mutagenic agents such as peroxynitrite which react with DNA, causing genomic alterations like point mutations, deletions or rearrangements in proliferating cells. Epidemiological studies have also reported an association between chronic inflammation induced by infectious agents, such as *Helicobacter pylori*, *Schistosoma haematobium*, and the Hepatitis B and C viruses, and the development and progression of certain cancers, such as gastric, esophageal, colorectal, liver, pancreatic, bladder and lung cancers [65].

Another cause of chronic inflammation that can promote cancer is obesity due to hypertrophy of adipose tissue and the associated areas of hypoxia [11]. This process causes tissue damage and a local inflammatory response due to the production of platelet-derived growth factor, transforming growth factor beta, complement factors that stimulate neutrophil chemotaxis, monocyte chemoattractant protein 1 (MCP-1), IL-1β and TNFα [11,66]. In addition, MCP-1 may contribute to the proliferation of macrophages in adipose tissue and, together with the infiltration of lymphocytes and their pro-inflammatory mediators, increase tumor growth. In fact, several cancers that arise near adipose tissue (e.g., breast cancer) have been reported to be associated with obesity [66].

Last but not least, the gut microbiota represents a third element that plays a role in this complex inflammatory phenomenon. The gut microbiota, in conjunction with diet, can contribute to the development of cancer, particularly colon and rectal cancer [67]. The small intestine is responsible for our body’s absorption function and some nutrients, such as carbohydrates, protein residues and bile acids, can pass from the small intestine into the large intestine. Excess nutrients, obesity or metabolic syndrome lead to intestinal dysbiosis. Changes in the diversity of the intestinal microbiota can cause carcinogenic substances, an impairment of the immunological properties of the intestinal epithelium and the development of stromal-cancer-associated adipocytes, which stimulate carcinogenesis [64].

These substances, known as “oncometabolites”, interact with the host cells. First, some primary bile acids are metabolized by Bacteroidetes and Bilophilia and converted into secondary bile acids, and these compounds eventually damage the membrane of enterocytes and generate ROS. As a result, they increase the inflammatory state. In addition, these secondary bile acids inhibit DNA repair systems, leading to the genetic instability of mutant cells [68,69]. Secondly, remnants of protein peptides and mucins are processed by bacteria into molecules—p-cresol, hydrogen sulfide (H_2_S), ammonia, nitrates, nitrites, etc.—which are toxic and increase the risk of cancer. For example, p-cresol is produced during the fermentation of tyrosine; it stimulates ROS production, which promotes tumor growth and increases cell detachment. This leads to the destruction of the mucosal barrier [68,70]. In summary, one’s diet influences the composition of the microbiota; some studies confirm that colorectal cancer patients have increased bacterial species with pro-inflammatory and cancer-promoting properties, such as *Fusobacterium*, *Clostridium*, *Desulfovibro*, *Prevotella* and *Enterococcus*. These conditions can lead to dysbiosis and increase the risk of cancer development [67]. In addition, bacterial populations such as *E. Coli*, *Fusobacterium nucleatum* and *Bacteroides fragilis* are capable of producing their own toxins that cause DNA damage and promote tumor growth. For example, *E. coli* produces colibactin, a genotoxin that forms cross-linking structures in DNA that promote the growth of colon tumors by activating the senescence program [71].

As mentioned above, obesity can lead to changes in intestinal permeability and dysbiosis which may increase the risk of colorectal cancer, as metabolic endotoxemia along with adipose tissue inflammation produce proinflammatory cytokines [1,16,67]. Sánchez-Alcoholado and coworkers [67] investigated the composition of the gut microbiota in obese and non-obese colorectal cancer patients compared to non-obese healthy patients. In both groups, they analyzed the following: zonulin (a marker of intestinal permeability), TMAO (a CVD-related microbial metabolite), IL-1β (proinflammatory factors) and IL-10 (anti-inflammatory factors) levels. Obese people with colorectal cancer had higher levels of zonulin, TMAO and IL-1β and lower levels of IL-10 than non-obese colorectal cancer patients and healthy controls. Obese colorectal cancer patients were also found to have a microbiota consisting of *Enterobacteriaceae* and *Streptococcaceae* such as *E. coli* and *Streptococcus bovis* compared to the other two groups described above. These bacterial species are known to cause an inflammatory state by altering the intestinal barrier and producing IL-1β and TMAO, which correlate with the development of colorectal cancer [67].

Inflammatory bowel diseases (IBD), comprising Crohn’s disease and ulcerative colitis, are chronic inflammatory conditions affecting the gastrointestinal tract, influenced by a complex interplay of genetic, environmental, immunological and microbial factors. The relationship between IBD and the gut microbiome, as well as dietary influences, plays a crucial role in the development, progression and management of these diseases [72,73]. The gut microbiome, including bacteria, fungi, viruses and other microbes, plays a fundamental role in gut health and immune regulation. In individuals with IBD, alterations in the gut microbiome’s composition and diversity have been observed. This dysbiosis may contribute to inflammation and immune system dysregulation, exacerbating IBD symptoms. Likewise, dysbiosis can trigger an inappropriate immune response, leading to the chronic inflammation found in IBD. Disruptions in the intestinal barrier function, caused by dysbiosis and inflammation, allow for the penetration of harmful bacteria or microbial products, further stimulating immune responses and perpetuating inflammation. Diet is a significant factor influencing the gut microbiome and IBD. Certain dietary components, such as a high intake of processed foods, saturated fats and sugar and a low intake of fiber-rich foods, have been associated with an increased risk of developing or exacerbating IBD symptoms, potentially influencing disease severity and progression. Diets rich in fiber from fruits, vegetables and whole grains have been associated with a lower risk of IBD and may help manage symptoms [74].

## 8. Genetics and Individual Susceptibility to Postprandial Dysmetabolism

The relationship between genetics and postprandial dysmetabolism represents a complex interplay influenced by enzymatic activity and genetic variations in metabolic pathways. Postprandial dysmetabolism encompasses disturbances in glucose and lipid metabolism following a meal. Understanding its genetic foundations is vital in elucidating individual responses to dietary intake [34,48,52]. Enzymes pivotal in nutrient breakdown and utilization are encoded by genes susceptible to polymorphisms. Gene polymorphisms significantly contribute to the intricate landscape of postprandial dysmetabolism, influencing an individual’s response to nutrient intake and subsequent metabolic outcomes. Gene variations implicated in postprandial dysmetabolism include those associated with glucose metabolism, such as amylase (AMY1), insulin (INS), glucokinase (GCK) and glucose transporter proteins (GLUTs) [75]. These polymorphisms have demonstrated effects on insulin sensitivity and glucose uptake, thereby contributing to an inconsistency in postprandial glycemic responses. Likewise, in lipid metabolism, genetic variants within genes encoding lipoprotein lipase (LPL), adiponectin, apolipoprotein E (APOE) and peroxisome proliferator-activated receptor gamma (PPARG) have been associated with alterations in postprandial lipid profiles. These variations influence processes like lipid clearance, triglyceride metabolism and cholesterol handling, thereby impacting post-meal lipid concentrations and metabolic trajectories. Moreover, polymorphisms within genes that are linked to appetite regulation, including leptin (LEP) and ghrelin (GHRL), have been proposed to modulate satiety responses, potentially influencing eating behaviors and subsequent postprandial metabolic alterations. Further comprehension of the intricate role of gene polymorphisms in postprandial dysmetabolism would enable personalized interventions. Integrating this knowledge into tailored strategies for dietary recommendations and lifestyle modifications may offer possibilities to mitigate adverse postprandial metabolic effects [37,38]. The emerging field of nutrigenetics emphasizes the interaction between genetics and diet, aiming to personalize nutrition recommendations based on an individual’s genetic makeup [38,76]. However, the impact of gene polymorphisms on postprandial dysmetabolism extends beyond individual genetic variations, since interactions with environmental factors and lifestyle habits, along with the complex interplay among various genetic determinants, contribute to heterogeneous metabolic outcomes [76].

## 9. Conclusions and Future Directions

Modern dietary patterns characterized by an excessive intake of processed foods and refined sugars and inadequate consumption of whole foods have been associated with a heightened risk of chronic diseases, particularly those related to metabolic dysregulation. These dietary habits contribute significantly to dysmetabolism, encompassing a spectrum of metabolic disorders such as insulin resistance, dyslipidemia, obesity and type 2 diabetes mellitus, elevating one’s susceptibility to cardiovascular diseases, cancer and metabolic syndrome.

The promotion of human health through a balanced diet is vital. Recommendations for an optimal diet regimen conducive to human health include the consumption of a diverse array of whole foods comprising fruits, vegetables, whole grains, lean proteins (including legumes and fish), nuts and seeds. These whole food sources provide essential nutrients, dietary fiber, antioxidants and phytochemicals, which are pivotal for maintaining overall health and mitigating metabolic risks. In addition, the restriction of processed foods and refined carbohydrates is crucial since these dietary components have been implicated in exacerbating insulin resistance, weight gain and metabolic dysfunction.

Addressing dysmetabolism diseases requires a multifaceted therapeutic approach, including lifestyle modifications and dietary interventions. In specific cases, medications targeting metabolic disorders, such as insulin sensitizers, lipid-lowering agents or glucose-lowering medications, may be necessary. Indeed, a balanced diet comprising whole foods, along with lifestyle modifications and, if needed, medical intervention, plays a pivotal role in preventing and treating dysmetabolism diseases and promoting overall human health.

Inflammation is a biological response of the body and a normal part of host defense to restore homeostasis and prevent the loss of tissue function. The acute postprandial inflammatory response can be considered a physiologic phenomenon; indeed, IL-1β produced postprandially in intestinal macrophages stimulates insulin release, which promotes peripheral glucose disposal and stimulates the uptake of glucose by immune cells. Similarly, the inflammatory response triggered by a high–fat meal could be a physiological phenomenon, as the presence of large numbers of chylomicrons in the bloodstream can activate leukocytes to remove them and trigger a physiological inflammatory cascade. However, if this process becomes unbalanced, leukocyte activation can lead to endothelial dysfunction and atherosclerosis.

Therefore, the acute postprandial inflammatory response following food intake appears to be a protective response that counteracts the effect of macronutrients. However, if the immune system is constantly activated, this may promote the development of low-grade inflammatory diseases. Epidemiologic studies in humans have identified a number of potential dietary components that have anti-inflammatory and pro-inflammatory components. An unbalanced dietary composition or malnutrition high in pro-inflammatory factors that activate the innate immune system may act as a trigger for chronic systemic low-grade inflammation, which may be associated with the development of cancer and chronic diseases.

Inflammation can therefore be portrayed as “Dr. Jeckyll and Mr. Hyde”: it serves as a defense but also leads to metabolic disease. However, since most chronic inflammatory diseases are associated with diet, dietary modifications with a balanced healthy diet, hypocaloric nutritional interventions and high levels of anti-inflammatory food components could prevent chronic inflammatory diseases and cancer.

In cancer, according to some authors, the cells of the body’s immune system produce chemokines and cytokines that have pro-inflammatory, tumor-promoting effects and promote angiogenesis, DNA damage and a tumor microenvironment that facilitates neoplastic invasion. However, native cells of the immune system can also produce chemokines and cytokines that may have anti-inflammatory and antitumor effects. A challenge in the future may be to evaluate whether polymorphisms in immune-related genes that regulate inflammatory processes play a role in promoting an imbalance in favor of chemokines and cytokines with pro-inflammatory and anti-tumor effects. Further research on immune regulation is needed to investigate how innate immune cells can be reprogrammed from a pro-inflammatory and tumor-promoting phenotype to an anti-inflammatory and tumor-fighting phenotype.

This duality may allow future research to focus on how innate immune cells can be modified to suppress levels of pro-inflammatory and tumor-promoting cytokines while increasing anti-inflammatory and tumor-fighting cytokines. Translational research studies should be conducted in order to explore potential benefits for human health.

The intricate interplay between genetic predispositions and environmental influences orchestrates an individual’s metabolic response to dietary stimuli. Understanding the genetic determinants of postprandial dysmetabolism holds promise for developing precise nutrition strategies. This knowledge can facilitate personalized interventions to manage and prevent metabolic disturbances associated with imbalanced dietary patterns.

## Figures and Tables

**Table 1:** Postprandial dysmetabolism and main mechanisms leading to *Diabetes mellitus* type 2, cardiovascular diseases and tumor development (IL: interleukin, CCL: C-C motif ligand, TNF: tumour necrosis factor, ROS: reactive oxygen species).

Etiology	Mechanisms	Effects	Disease
Postprandial dysmetabolism: hyperglycemia and hyperlipidemia	Production of pro inflammatory cytokines (IL-1β, TNFα, CCL-2) Infiltration of immune cells in islets of Langerhans β cell failure	Inhibit insulin signaling in liver and adipose tissue Increased ubiquitination and degradation of insulin. Transcription of inflammatory genes.	*Diabetes mellitus* type 2
	Electron chain overload in muscle and adipose tissue mitochondria Adipose tissue and connective tissue cells produce and release pro-inflammatory cytokines (TNFα, IL-1β). Increased activity of platelets.	Increased ROS production Increased expression of adhesion molecules in the endothelium Increased platelet aggregation	Cardiovascular diseases
	Hypertrophy of adipose tissue with areas of hypoxia Intestinal dysbiosis: Bacteria produce onco-metabolites that damage enterocytes Increased ROS production Bacteria produce toxins that contribute to cell damage	Tissue damage due to local inflammatory response Infiltration of lymphocytes. DNA damage	Tumor development

## Data Availability

No new data were created or analyzed in this study. Data sharing is not applicable to this article.
